# Investigating enhancement of learning and memory following supplementation with Juice Plus^+^® Omega in an adolescent population

**DOI:** 10.1007/s00394-026-04012-9

**Published:** 2026-06-29

**Authors:** Lynne Bell, Jessica Eastwood, Manfred Lamprecht, Claire M. Williams

**Affiliations:** 1 School of Psychology & Clinical Language Sciences, University of Reading, Reading, UK; 2 The Juice Plus+ Science Institute, Collierville, TN USA

**Keywords:** Omega fatty acids, Cognition, Mood, Emotion regulation, Adolescence

## Abstract

**Purpose:**

Omega fatty acids (FA) have been shown to benefit cognition during infancy and adulthood. However, adolescence remains under investigated, despite being a critical period for development of executive functions and emotion regulation. The current objective was to investigate the impact of daily treatment with a proprietary omega-FA blend on cognitive performance, mood, and emotion regulation in healthy adolescents aged 13–14 years.

**Methods:**

Using a parallel, double-blind, placebo controlled design, participants were randomly allocated to consume the omega blend (2 capsules/day providing 925 mg blend of algae-derived omega 3-, 7-, and 9-FAs) or a placebo (2 capsules/day of MCT oil) for 16 weeks. Episodic memory, executive function, mood, emotion regulation, EEG measures, and omega-3 index (O3I) were recorded at baseline and following 16 weeks of intervention.

**Results:**

O3I increased significantly only in the omega blend group, indicating compliance with the intervention and improved O3I status. Improvements were observed for immediate word recall and delayed word recall aspects of episodic memory in the omega blend group only. Significantly faster reaction times were also observed on an attention network executive function task in the omega group. Alongside cognitive benefits, changes in EEG activity were observed, including increased N200 ERP deflections during 0-back task performance, and reduced PSD activity during sustained attention and at rest.

**Conclusion:**

Combined, these cognitive and physiological findings suggest that an omega-FA blend may support cognitive development in healthy adolescents aged 13 & 14, potentially through facilitation of brain maturation and more efficient allocation of neural resources.

**Clinical Trial number:**

ClinicalTrials.gov ID: NCT05581108. Registration date: 11/10/2022.

**Supplementary Information:**

The online version contains supplementary material available at 10.1007/s00394-026-04012-9.

## Introduction

Diet represents one of the most important lifestyle factors that can strongly influence cognitive functioning. While a food-first approach is recommended, supplementing the diet with certain nutrients can be helpful in ensuring nutritional requirements are met, thereby supporting optimal health. Thus, a healthy diet supported by supplements where required is an essential factor for healthy brain function throughout life. Previous studies of omega-3 polyunsaturated fatty acids (PUFAs) intake have shown beneficial actions on a range of human health conditions. The influence of omega-3 PUFAs on cognition throughout the lifespan is particularly apparent, with beneficial effects documented to cognitive development in infants and children [1], and to cognitive performance in young adults [2], along with evidence for a slowing of age-related cognitive impairment in older adults [3]. While benefits of omega-3 PUFAs to cognitive function are not always apparent in supplementation trials [4–6], benefits have been shown in humans to visual attention, working memory and executive function aspects of cognition [1], including selective attention [2] in younger populations, and in episodic memory in older populations [3]. Monounsaturated fatty acids (MUFAs) such as omega-7, and omega-9 are also associated with cognition and are particularly known for their potential neuroprotective effects against cognitive decline [7], with animal models suggesting protective effects mainly on memory.

Despite the positive findings described above, there has been little consideration of the influence of omega-3 PUFAs or other MUFAs on cognition in healthy adolescence. Some previous research has noted positive associations between omega-3 intake and mental health in this age group [8, 9], and some work has investigated omega-3 benefits in ADHD or neurodevelopmental disorders [10]. However, healthy adolescence is characterized by profound brain development, with brain areas such as the prefrontal cortex continuing to mature into the late twenties [11]. In this period of brain development, the basis is laid for executive functions (e.g., shifting, updating, and short-term memory), alongside development of emotion regulation. Optimal development of the prefrontal cortex is very important, as the executive functions have been related to future academic achievements [12], and emotion regulation capabilities have been linked to better mental health [13]. Given that positive effects of omega-supplementation have been observed across these cognitive domains in other older and younger populations, it seems likely that adolescents would also benefit from supplementation. Omega supplementation has also been associated with increased neural efficiency [14], which suggests a possible mechanism of action by which omega supplementation might benefit cognitive function. Therefore it is possible that omega-FAs may facilitate improved neural and cognitive function in adolescents during this critical period of cortical development. The positive effects from omega-3 PUFAs or other MUFAs on cognition via neural efficiency, if translated to healthy adolescents, would therefore be of clear practical and theoretical importance, particularly in an academic context.

There are many proprietary omega-3 supplements on the market. Some supplements combine omega-3 with additional fatty acids such as omega-6 and omega-9 to provide a more balanced fatty acid profile. However, the intake ratio for various fatty acids has been shown to be important. For example, if the ratio of omega-6 to omega-3 is too high there may be risks for increased inflammation [14], although the extent of this relationship is still not fully understood [15]. Typically, the Western diet is already high in omega-6 and so further supplementation may not be needed [14]. Juice Plus+Essentials OMEGA+Blend contains a mixture of omega-3, -7, and -9 derived from plant-based sources, and has already shown effectiveness in improving fatty acid indices in healthy subjects [16]. Therefore, we investigated the impact of 16 weeks daily treatment with the same blend, on cognitive performance, mood, and emotion regulation in healthy adolescents aged 13–14 years, with EEG recording to determine any changes in neural activity.

## Methods

### Design

**Fig. 1:**
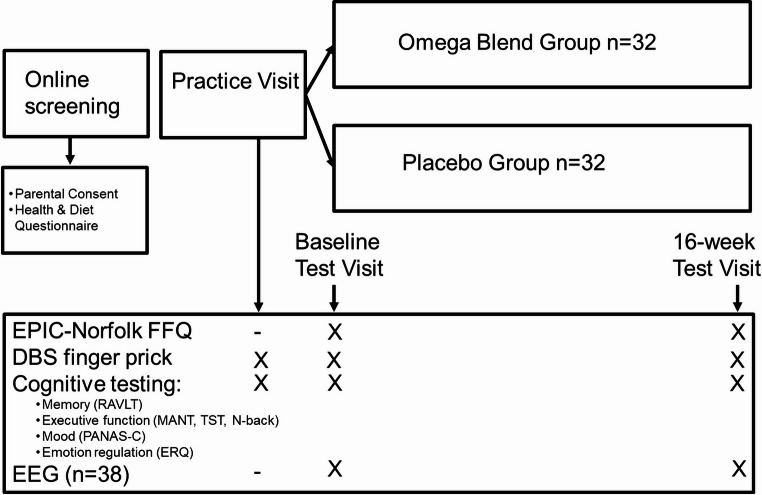
Study design

A between-groups chronic study design was used to examine the effects of omega blend on cognitive performance and mood in healthy adolescents aged 13 and 14 years. Participants were randomly allocated to consume either omega blend or placebo daily for 16 weeks. The study was conducted double-blind. Cognitive and EEG measures were taken at baseline prior to consumption of the intervention then again after 16 weeks. Finger prick samples were taken at baseline and 16 weeks to test for omega 3 index (as a measure of compliance and efficacy). An overview of the complete trial design is shown in Fig. 1.

### Participants

A priori power analysis was performed using GPower 3.1.9.7 to determine the number of participants required to achieve a statistical power of 0.8 with an alpha level of 0.05 for a repeated measures ANOVA, within-between interaction. Previously, effect sizes (Cohen’s d) of 0.28 and 0.32 have been reported for the effect of DHA and omega-rich nut interventions on episodic memory in young adults [17, 18]. Assuming the larger effect size of 0.32 (Cohen’s f 0.16), a sample size of 60 was deemed sufficient. To allow for a 10% attrition rate, the aim was to recruit 66 healthy adolescents (N = 33 per treatment), with the final aim of achieving a minimum n = 60. Subjects were considered eligible to participate if they met the criteria outlined in Table 1. Sixty-four participants were subsequently recruited.

**Table 1:** Eligibility criteria

Inclusion criteria	Exclusion criteria
13–14 years old	Psychological or psychiatric disorders
Not more than 3 servings of fruits or vegetables per day	ADHD or dyslexia diagnosis
Not more than 2 servings of fish per week	Fruit/vegetable or histamine intolerance
Fluent in English	Use of medications, dietary supplements that may impact study outcomes
Adequate visual & auditory acuity to perform the cognitive tasks	Conditions altering absorption of nutrients (e.g. celiac disease)
Normal BMI for age	Antibiotic use within the last 3 months
	Adherence to any specific diets that may impact study outcomes (i.e. vegetarian, paleo etc.)

### Interventions

Both the treatment and placebo interventions were taken in capsule form (2 capsules) daily with breakfast. The composition of the omega blend treatment is shown in Table 2. The placebo contained MCT oil (nutritional breakdown not specified). Treatments were matched for appearance and provided to researchers in opaque screw-cap bottles labelled A or B. Upon enrolment, participants were randomised to receive either A or B using block randomisation, meaning per every 10 participants enrolled 5 were randomised to group A and 5 to group B. As such, both participants and researchers were blind to treatment allocation.

**Table 2:** Nutrition information for Juice Plus^+^ Essentials OMEGA^+^ Blend

Nutrient	Per 2 capsules
Total omega fatty acids of which:	925 mg
Omega -3 fatty acids of which:	375 mg
DHA	175 mg
EPA	100 mg
Omega-5 + 7 fatty acids	250 mg
Omega-6 + 9 fatty acids	250 mg

### Assessments

#### Rey auditory verbal learning task (RAVLT)

This episodic memory task was a multi-trial word list learning task with immediate and delayed free recall, and delayed recognition components [19–21]. Participants listened to a recording of fifteen words and were immediately asked to verbally recall as many as possible in one minute. The same word list (List A) was repeated a further four times, each followed by a recall period (R1 to R5). Another unrelated list of 15 distractor words was then introduced (List B). Participants verbally recalled List B (RB) before again recalling List A (R6). After a delay of approximately 15 min, during which time the remaining cognitive tasks were completed, participants again verbally recalled List A (R7), then performed a visual recognition task selecting List A words from a list of 50 words also containing the 15 List B distractors and 20 previously unseen words. Different word lists, matched for concreteness and familiarity, were used at each test session and counterbalanced between groups [20, 21]. The dependent variables were immediate word span (R1), total acquisition (sum of trials R1-R5), final acquisition (R5), proactive (R1-RB) and retroactive interference (R5-R6), learning (R5-R1) and delayed recall (R7) and recognition [19], as well as a modified measure of acquisition (trials 1–5 with recall trial number as factor in statistical model). Total acquisition represents the primary outcome measure in the current study. All other cognitive and mood measures are secondary, while EEG is treated as exploratory.

#### Modified attention network task (MANT)

This executive function task combines the Eriksen Flanker task [22] with the Posner spatial cueing task [23] to investigate multiple facets of attention: alerting, orienting, and executive attention [24]. Participants viewed blocks of arrows presented on screen in rapid succession and indicated the direction of the arrow closest to a central fixation point with a key press (left or right arrow). The target arrow was either flanked by arrows pointing in the same (congruent) or opposite (incongruent) direction or was not flanked at all [21, 25]. On selected trials, spatial cues were introduced immediately prior to the appearance of the arrows. Task load was further manipulated by increasing or decreasing the number of flanking arrows across multiple trials. The dependent variables were average reaction time (ms) and accuracy (% correct expressed as a decimal). For this task, cognitively demanding trials were considered to be incongruent, high load trials as described above.

#### Task switching task (TST)

This executive function task employed a modified version as described in Miller et al. [26]. Participants viewed eight equally spaced radii of a circle displayed in such a way that there were four equally spaced segments above and below a bold line. A stimulus digit selected from between 1–9 (excluding 5) appeared in each segment in turn in a clockwise direction. Each digit was displayed for a duration of 1000 ms. Inter-stimulus interval was 500 ms. Dependent on whether the stimulus was in the segments above or below the bold line, participants performed different tasks. If the number was above the bold line, participants discerned whether the stimulus was odd or even by pressing the relevant response key, whereas if the number was below the bold line, participants discerned whether the number was higher or lower than 5, again by pressing the relevant key. The task switched every 4 trials. The dependent variables were average reaction time (ms) and accuracy (% correct expressed as a decimal). For this task, cognitively demanding trials were considered to be the switch trials, which required the volunteer to change from performing one task to the other as described above.

#### 0-back task

This task was used primarily for EEG data collection as this simplest form of the n-back task which requires sustained attention for target detection but does not introduce the working memory element is known to elicit clear N200 and P300 peaks for ERP analysis [27]. During this attention task, participants viewed a sequence of letters and were required to differentiate targets (letter z) from non-targets (all other letters) using yes (b) and no (n) keys on the keyboard, respectively. The dependent variables were task accuracy (%) and reaction time (ms), which were analysed for both target and non-target trials (n = 40 trials and n = 120 trials, respectively).

#### Positive and negative affect schedule for children (PANAS-C)

During the PANAS-C [28] mood questionnaire, participants rated 30 mood adjectives (15 positive and 15 negative) on a scale of 1–5. Dependent variables were composite positive affect and negative affect scores obtained by summing responses to the positive or negative PANAS items, respectively. Total self-reported rating score for overall positive and negative affect was out of 75. These subjective mood variables were recorded at the beginning of the test battery.

#### Emotion regulation questionnaire (ERQ)

The ERQ questionnaire [29] included a series of statements about emotions that participants were asked to rate their agreement with, on a scale of 1 (strongly disagree) to 7 (strongly agree). Dependent variables include a Reappraisal score and a Suppression score.

#### EEG

All participants were tested in our dedicated lab within the Reading University Centre for Integrative Neuroscience and Neurodynamics at baseline and 16 weeks. EEG data was recorded using the Brain Products ActiCap EEG system with 16-channel active electrode caps at a sampling rate of 5 kHz and sampling interval of 200 ms. Electrodes 1 – 16 were placed as follows, in addition to a left VEOG: Fz, F3, FC5, FC1, C3, T7, Pz, P3, Oz, Cz, P4, T8, C4, FC2, FC6, F4. The reference electrode was placed at FCz and the ground at FPz. Correct fitting of the EEG cap is essential for the accurate positioning of electrodes. Head circumference was used to determine appropriate cap size, and the mid-points between the nasion and inion and between the left and right ears were used to ensure the correct positioning of electrode Cz. Electrode positioning on the cap was in accordance with the international 10–20 system. To ensure an adequate EEG signal, testing commenced once impedances were < 10 kilo ohms. Volunteers were asked to keep as still as possible whilst the recording was taking place to avoid movement related artefacts.

EEG data collected during the 0-back task and during rest with eyes open and eyes closed were analysed to determine any difference in PSD (µV^2^/Hz) for delta, theta, alpha, and beta wavebands (lower frequency bands typically observed in children) between the omega blend and placebo interventions during periods of sustained attention, active rest and passive rest, respectively. The dependent variable was mean activity (µV). Frequency bandwidths are associated with different cognitive processes. For example, alpha band changes during attentional tasks may be related to internal thought processes [30], whilst delta band activity may be associated with inhibition of the processing of sensory information as required for optimal internal concentration [31]. Theta bandwidth is also thought to be associated with memory encoding [32], and cognitive control [33, 34]*.* In childhood and adolescence reductions in delta, theta, alpha, and beta have previously been associated with brain maturation [35]. PSD was determined for frontal (Fz, F3, F4) and parietal (Pz, P3, P4) brain locations by averaging mean activity across electrodes in these regions, both areas of known change during adolescent brain development [35].

In addition to PSD, ERP analysis was performed to determine any differences between treatment conditions in parietal amplitude (mean of highest voltages of the deflection from peak to trough, µV), and latency (ms) of N200 and P300 peaks during performance of the 0-back task. The N200 is a negative deflection occurring at approximately 180-325 ms following stimulus presentation whilst the P300 is a positive peak occurring approximately 250-400 ms after stimulus presentation [36, 37]. Considering both occur after 100 ms of stimulus presentation they are thought to reflect the cognitive processing of information as opposed to sensory processing which occurs typically within 100 ms of stimulus presentation [36], therefore reflecting attention-based stimulus evaluation [37]. ERP data was collected for both target and non-target trails during the 0-back task, given that each represents different cognitive processes which are of interest in relation to treatment [38]. Brain Vision Analyser software was used to convert the raw EEG signal into PSD and ERP data for statistical analysis. An overview of the analysis pipeline is described below:

Prior to determination of PSD and ERPs, the raw EEG signal underwent pre-processing using high and low-pass filters (0.1 Hz and 64 Hz respectively) with a notch of 50 to remove potentially erroneous frequencies as well as automatic detection and correction of artefacts related to eye movement using Brain Vision Analyser’s automatic Ocular Correction Independent Components Analysis. To obtain PSD data during a period of sustained attention (0-back task) and during rest (eyes open, and eyes closed), following the pre-processing stages described above, the signal was segmented using markers according to the task of interest such that the signal from the start until the end of the task and hence across all trials was used in the analysis. The signal was then further segmented according to 2 s equal segments with a sampling rate of 256 Hz. Mean activity of PSD for delta (0.5–3.5 Hz) and theta (3.5–7.5 Hz), alpha (7.5–12.5 Hz), and beta (12.5-30 Hz) bandwidths was determined using Fast Fourier Transform and exported for statistical analysis. To obtain the ERP data, following ocular correction, the signal was segmented into time-based epochs using markers relevant to the task of interest. A baseline correction using 200 ms of signal prior to the presentation of the stimulus was then applied. Signal for each electrode was then averaged across trials for every subject at each timepoint. Automatic peak detection was then carried out on those subject averages for each electrode at each timepoint using reference ranges guided by the literature[37]. Specifically, the limits of detection for the N200 and P300 components were set at 180-325 ms and 250-400 ms respectively. Peak amplitude and latency for the N200 and P300 for all trials in the 0-back taskwere then exported for statistical analysis.

#### Compliance

Dried blood spot samples obtained from finger pricks were analysed for omega 3 index (a measure of omega 3 levels in cell membranes of erythrocytes), before and after the 16-week intervention.

### Procedure

The randomised, placebo-controlled, double-blind, 2-arm study design is illustrated in Fig. 1. Following online screening for eligibility, participants attended three separate visits to the University of Reading Psychology department; a practice visit and two test visits. Visits were scheduled after school from 4 to 6 pm. At the practice visit, participants were trained how to perform the cognitive tasks, and were asked to swallow an example capsule (to check capsule swallowing ability), and were measured for an EEG cap if they also wished to take part in EEG. For 12 h prior to each test visit, participants followed diet and lifestyle restrictions, which involved adherence to a low PUFA and low polyphenol diet. Volunteers were asked to recall everything they consumed during the 12 h before attending the baseline visit and were asked to consume the same foods at the same times prior to their 16-week visit. Compliance was monitored using a second recall performed at the 16-week visit. Parents were also asked to complete the EPIC-Norfolk food frequency questionnaire [39] on behalf of their child at the beginning and end of the study, to provide a measure of habitual diet and to check whether any dietary changes occurred during the intervention period. The computerised cognitive test battery consisting of cognition and mood measures was performed first followed by EEG recording for a subset of participants. The order of cognitive and mood measures was as follows: AVLT (immediate recall), MANT, Switching Task, AVLT (delayed recall and recognition), and PANAS-C, followed by 0-back (with or without EEG recording). Dried blood spot samples were taken at the end of each visit. Participants received a 16-week allocation of capsules to take daily between the baseline and 16-week visits.

### Statistical methods

All data were analysed using R (version 4.2.3).

Regarding the cognitive endpoints, for all RT data, only correct responses were included in mean values. For all behavioural and EEG data, outliers were identified for each Time × Treatment condition using Tukey’s IQR method, where values above Q3 + 3 × IQR or below Q1 – 3 × IQR were considered outliers and removed from the dataset prior to statistical analysis. Where relevant, condition or trial type was also taken into account when identifying outliers. Mixed ANOVA was used to analyse all dependent variables with time and treatment included as factors. Other factors were added to the statistical model where relevant i.e. congruency and cognitive load for the MANT, and trial type for the TST. Post-hoc pairwise comparisons were used to investigate any significant effects of treatment, time, or treatment x time interactions. A Bonferroni correction was applied to all multiple comparisons. Only trending or significant main effects and interactions along with their associated significant pairwise comparisons (*p* < 0.05) are reported. For the PSD and ERP analyses, data were analysed using ANOVA with Bonferroni corrected pairwise comparisons for each brain region of interest. Specifically, PSD data for all bandwidths were analysed for the 0-back task and at rest (eyes open and closed) in frontal and parietal regions. ERP analysis was performed on the 0-back data only. Regression analyses were performed to determine any relationship between O3I status, PSD activity, and cognitive function. Again, a Bonferroni correction was applied to correct for performing multiple analyses. Data of interest are presented in bar charts with between-subject error bars, while mean (M), standard error (SE) and p values can be found for all cognitive, mood and EEG data in Tables 5, 6, 7. Where outliers have been removed prior to analyses, the final N is denoted in the table. All data analyses, except exploratory regressions, were carried out prior to treatment unblinding.

## Results

**Fig. 2:**
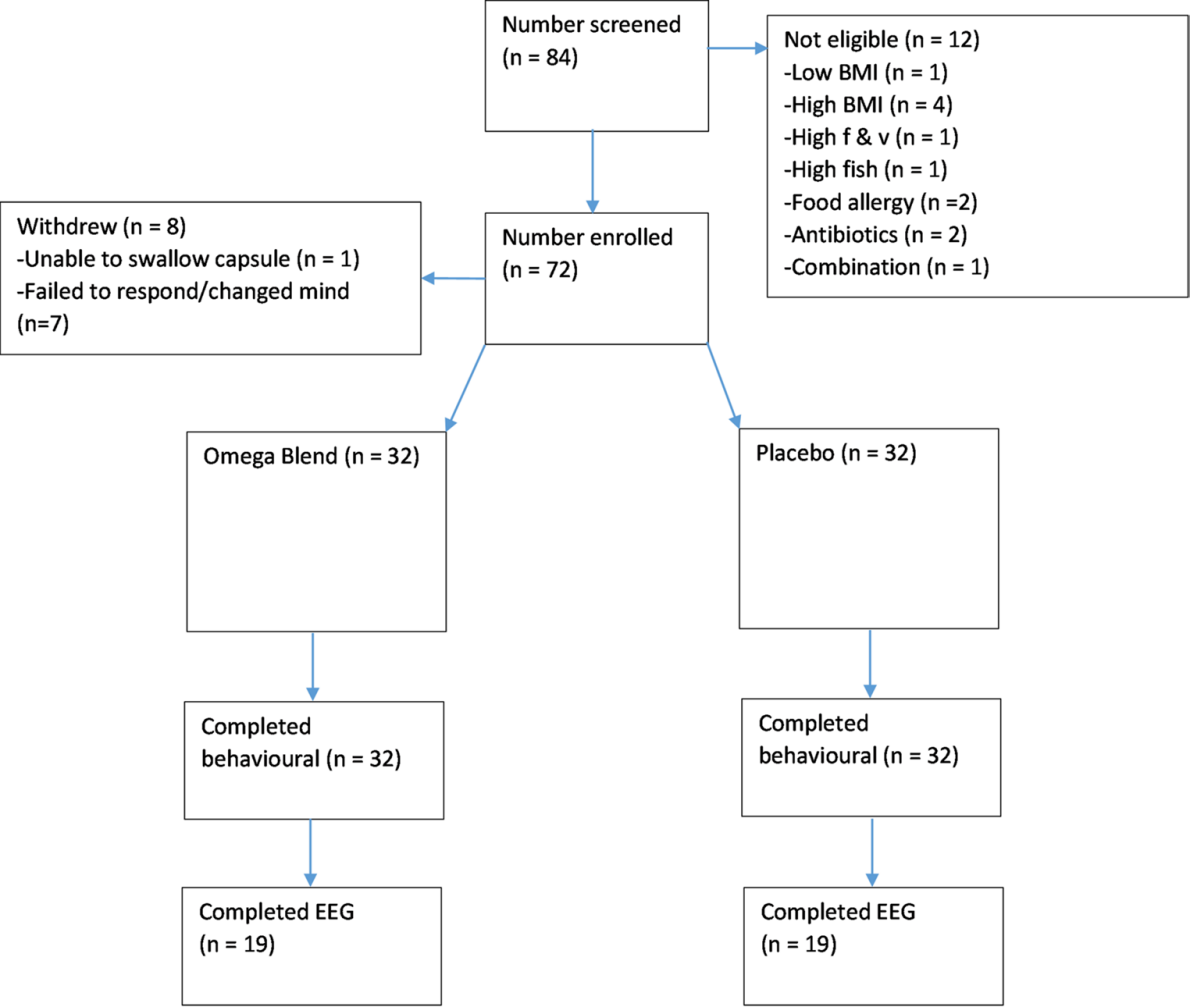
Consort diagram

Data was successfully collected for 64 participants as shown in the consort diagram (Fig. 2). Table 3 outlines sample characteristics at enrolment, for which there were no significant differences between groups with the exception of BMI where the omega blend group had a slightly larger BMI at enrolment compared to the placebo group. Subsequently, BMI was not found to correlate with O3I change or cognitive performance on the primary outcome measure, and therefore was not included as a covariate in statistical models.

**Table 3:** Participant demographics at enrolment

Characteristic	Omega Blend Group (n32)			Placebo Group (n32)			Group comparisons
	Mean	SD	Range	Mean	SD	Range	*p*-value (*p < .05)
Age (years)	13.59	0.50	13–14	13.38	0.55	13–14	.102
Gender (M:F)	15:17	N/A	N/A	13:19	N/A	N/A	.621
BMI (kg/m^2^)	19.58	1.87	16.11–23.92	18.56	1.92	15.60–22.53	.035*
Ravens Score (/15)	13.31	1.69	9–15	12.63	2.09	7–15	.154
O3I (%)	4.48	0.64	3.44–6.25	4.42	0.59	3.48–5.79	.660

Dietary ANOVA analysis revealed no main effects of time, treatment, or treatment x time interaction for any of the dietary outcome variables shown in Table 4 (*p* > 0.05, respectively). However, pairwise comparisons did highlight a small but significant difference in carbohydrate intake between the two treatment groups reported at the 16-week time point (*p* = 0.048), with the placebo group reportedly consuming 29.94 g more per day than the omega group (Table 4). Given that the omega group were observed to (non-significantly) reduce their carbohydrate intake by around 10 g per day between baseline and 16 weeks, it is possible that consumption of the omega blend might have exerted a greater satiating effect than the placebo oil, although there appears to be little support for this theory in previous literature [40]. However, as parents were completing the EPIC Norfolk FFQ on behalf of their children, there may be some inaccuracies in reporting. Indeed, the fruit and vegetable data suggest overestimation, given that participants reported consuming no more than 3 portions of fruit or vegetables per day via the diet and health questionnaire used at screening, whereas the FFQ data indicated greater than 3 portions daily. Despite issues with the accuracy of parental reporting, and with retrospective self-report in general, and aside from minor discrepancies in carbohydrate intake between treatment groups, participants appeared not to have broadly changed their overall diet during the study.

**Table 4:** Habitual diet reported by parents at baseline and 16 weeks

Dietary measure	Omega Blend Group (n32)				Placebo Group (n32)			
	Baseline		16-weeks		Baseline		16 weeks	
	Mean	SE	Mean	SE	Mean	SE	Mean	SE
Energy (kCal/day)	1619.24	94.21	1515.16	81.77	1727.64	89.38	1731.51	77.57
Carbohydrate (g/day)	193.55	11.77	183.21*	10.73	215.27	11.17	213.15	10.18
Protein (g/day)	71.94	4.24	66.12	3.40	73.75	4.02	72.31	3.23
Fat (g/day)	67.38	4.72	62.69	3.99	69.56	4.47	71.52	3.78
MUFA & PUFA (g/day)	33.99	2.32	31.90	2.07	35.18	2.20	36.47	1.97
Fish (portions/week)	0.96	0.15	1.13	0.15	1.22	0.14	1.31	0.14
Fruit & veg (portions/day)	4.09	0.38	3.80	0.33	4.09	0.36	3.73	0.31

Reported values are estimated marginal means; *significant between-groups difference *p* < 0.05

### O3I (Compliance)

**Fig. 3:**
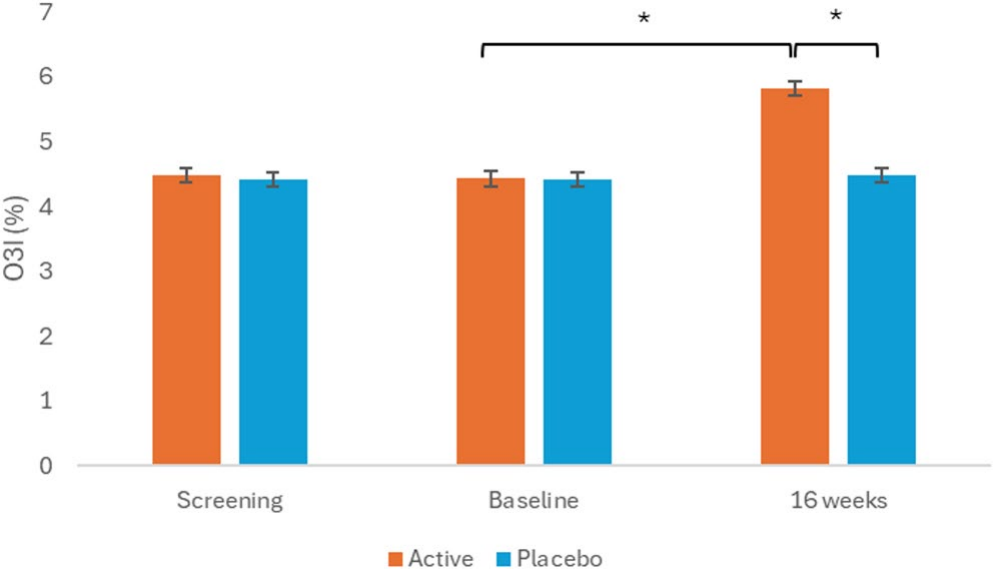
Mean O3I (%) following omega blend or placebo treatment. Values are estimated marginal means, *p < 0.05

O3I analysis revealed main effects of time [F(2,180) = 26.69, *p* < 0.001], treatment [F(1,180) = 26.27, *p* < 0.001], and a significant time x treatment interaction [F(2,180) = 22.20, *p* < 0.001]. Pairwise comparisons highlighted significant increases between baseline and 16 weeks in the omega blend group only (*p* < 0.001; Cohen’s d = 2.21), resulting in a significantly higher value for the omega group compared to the placebo group at 16 weeks (*p* < 0.001; Cohen’s d = 2.13)(see Fig. 3). As a further measure of compliance, participants were requested to return any unused capsules at the end of the study. Two omega group participants and 3 placebo group participants omitted to return their unused capsules as requested. Among the other participants, compliance was generally good (placebo: M 93.06%, SD 8.52%; omega blend: M 91.07%, SD 10.79%), with no significant difference in compliance between groups (*p* = 0.381). On average, the placebo group missed 15 capsules (equivalent to 7.5 out of 112 days), while the omega blend group missed 20 capsules (equivalent to 10 out of 112 days). Combined, the compliance data and O3I data suggest that omega blend treatment was well tolerated in this demographic, and effective in facilitating physiological change, with mean O3I levels increasing from around 75th to 95th percentile based on age-related norms [41].

### Cognition

ANOVA output for all cognitive outcomes can be found in Table 5.

**Table 5:** Cognitive data – summary statistics from ANOVA analyses

Measure	N	Omega blend				Control						
		Baseline		16 weeks		Baseline		16 weeks		ANOVA Effects [(df) = F, p-value]		
		Mean	SE	Mean	SE	Mean	SE	Mean	SE	Treatment	Time	Treatment x Time
*AVLT recall (out of 15):*												
Immediate word span	64	6.25	0.27	6.72	0.27	6.19	0.27	6.69	0.27	(1,124) = 0.030, .864	**(1,124) = 3.164, .078†**	(1,124) = 0.003, .954
Total acquisition (out of 75)	64	48.90	1.42	53.10	1.42	50.00	1.42	51.40	1.42	(1,124) = 0.031, .861	**(1,124) = 3.815, .053†**	(1,124) = 0.932, .336
Final acquisition	64	12.10	0.36	12.90	0.36	12.00	0.36	12.30	0.36	(1,124) = 0.910, .342	(1,124) = 2.437, .121	(1,124) = 0.752, .387
Proactive interference	64	0.34	0.40	0.69	0.40	0.78	0.40	0.72	0.40	(1,124) = 0.3338, .562	(1,124) = 0.122, .728	(1,124) = 0.254, .615
Retroactive interference	64	1.81	0.29	1.53	0.29	1.34	0.29	0.97	0.29	**(1,124) = 3.093, .081†**	(1,124) = 1.253, .265	(1,124) = 0.026, .873
Learning	64	5.81	0.38	6.22	0.38	5.84	0.38	5.59	0.38	(1,124) = 0.599, .441	(1,124) = 0.041, .839	(1,124) = 0.731, .394
Delayed recall	64	9.91	0.50	11.41	0.50	10.16	0.50	10.75	0.50	(1,124) = 0.164, .686	(1,124) = 4.363, .039*	(1,124) = 0.817, .368
*AVLT delayed recognition:*												
Accuracy (out of 15)	63	12.70	0.30	13.40	0.30	12.50	0.30	13.10	0.30	(1,122) = 0.702, .404	(1,122) = 4.440, .037*	(1,122) = 0.019, .890
*MANT (Congruency):*												
All Trials: Accuracy (%)	50											
Congruent		0.98	0.01	0.98	0.01	0.98	0.01	0.98	0.01	(1,988) = 0.113, .736	(1,988) = 0.228, .633	(1,988) = 0.793, .373
Incongruent		0.90	0.01	0.90	0.01	0.92	0.01	0.90	0.01			
No congruency		0.98	0.01	0.98	0.01	0.98	0.01	0.97	0.01			
All Trials: RT (ms)	61											
Congruent		502	7.39	481	7.39	487	7.51	475	7.51	(1,1208) = 1.969, .161	(1,1208) = 15.381, < .001*	(1,1208) = 0.686, .408
Incongruent		566	7.39	543	7.39	561	7.51	549	7.51			
No congruency		494	10.45	469	10.45	480	10.62	457	10.62			
*MANT (Load):*												
All Trials: Accuracy (%)	48											
High		0.94	0.01	0.95	0.01	0.94	0.01	0.95	0.01	(1,948) = 0.791, .374	(1,948) = 0.350, .554	(1,948) = 0.005, .942
Medium		0.95	0.01	0.94	0.01	0.95	0.01	0.95	0.01			
Low		0.98	0.01	0.98	0.01	0.98	0.01	0.98	0.01			
All Trials: RT (ms)	62											
High		540	7.78	517	7.87	532	8.00	520	8.00	(1,1208) = 1.733, .188	(1,1208) = 13.540, < .001*	(1,1208) = 0.604, .437
Medium		528	7.87	508	7.87	516	8.00	504	8.00			
Low		494	11.13	469	11.13	480	11.32	457	11.32			
*Switching task:*												
All trials: Accuracy (%)	64	0.80	0.02	0.81	0.02	0.81	0.02	0.81	0.02	(1,124) = 0.062, .804	(1,124) = 0.186, .667	(1,124) = 0.010, .921
All trials: RT (ms)	64	628	9.86	619	9.86	614	9.86	612	9.86	(1,124) = 1.096, .297	(1,124) = 0.309, .579	(1,124) = 0.167, .683
*0-back:*												
Accuracy (%)	58	0.96	0.01	0.96	0.01	0.97	0.01	0.97	0.01	(1,112) = 2.628, .108	(1,112) = 0.224, .637	(1,112) = 0.552, .459
RT (ms)	64	517	11.00	493	11.10	482	11.10	480	11.00	(1,123) = 2.578, .111	(1,123) = 0.802, .372	(1,123) = 0.620, .433

Reported values are estimated marginal means; †non-significant trend 0.1 > *p* > 0.05, *significant effect *p* < 0.05

#### RAVLT

**Fig. 4:**
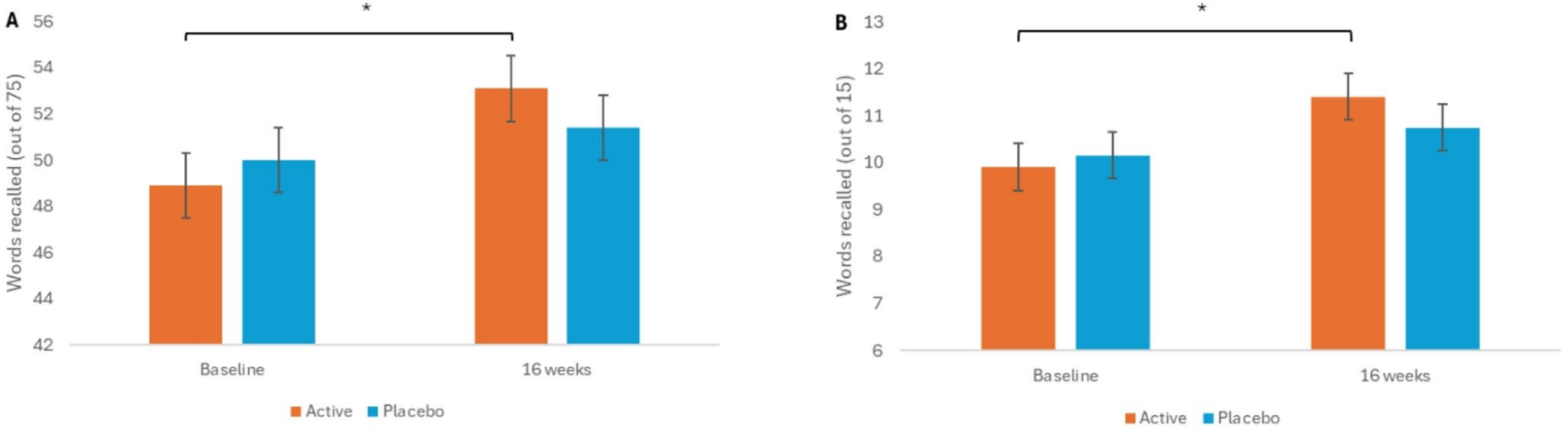
Mean RAVLT total acquisition scores (out of 75) (Panel A) and Mean RAVLT delayed recall scores (out of 15) (Panel B) following omega blend or placebo treatment. Values are estimated marginal means, * p < 0.05

For RAVLT there were no significant treatment-related effects for any of the outcome variables. However, near-significant or significant main effects of time were observed for the primary outcome measure total acquisition [F(1,124) = 3.815, *p* = 0.053] and delayed recall [F(1,124) = 4.363, *p* = 0.039], respectively. In both cases, pairwise comparisons revealed significant increases between baseline and 16 weeks in the omega blend group only (total acquisition *p* = 0.041, Cohen’s d = 0.52; delayed recall *p* = 0.036, Cohen’s d = 0.53), as shown in Fig. 4.

Trending or significant main effects of time were similarly observed for word span [F(1,124) = 3.164, *p* = 0.078] and delayed recognition [F(1,122) = 4.440, *p* = 0.037], but subsequent pairwise comparisons were non-significant. Likewise, a trend for a main effect of treatment was observed for retroactive interference [F(1,124) = 3.093, *p* = 0.081], but in the absence of any significant pairwise comparisons.

#### MANT

**Fig. 5:**
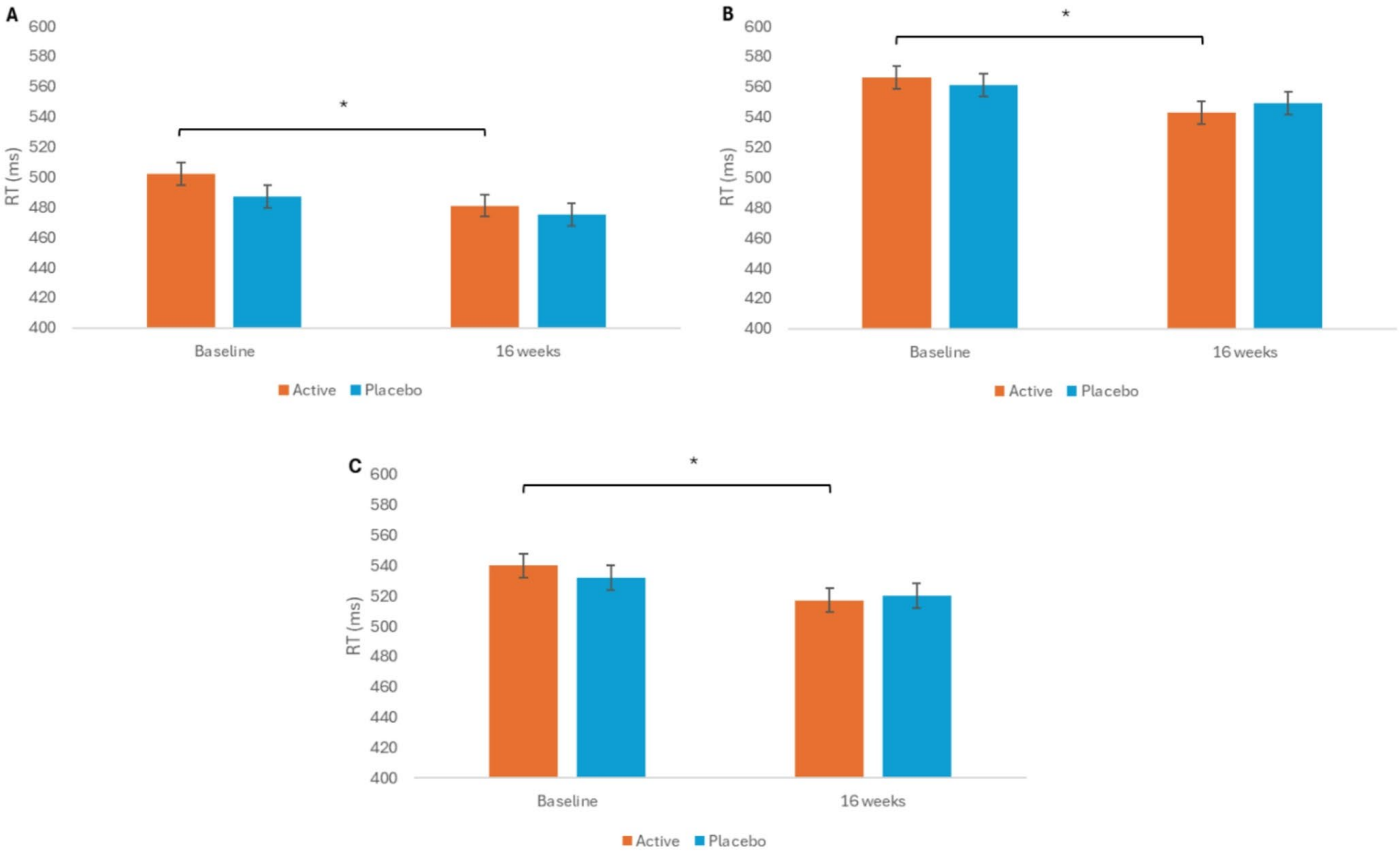
Mean MANT RTs (ms) following omega blend or placebo treatment during A: congruent, B: incongruent, and C: high load trials. Values are estimated marginal means, *p < 0.05

There were no significant treatment related effects for MANT accuracy. However, for MANT RT, ANOVA analysis including congruency as a factor revealed a significant main effect of time [F(1,1208) = 15.381, *p* < 0.001] and congruency [F(2,1208) = 114.539, *p* < 0.001]. Pairwise comparisons revealed a reduction in RT following omega blend, but not placebo, on both congruent (*p* = 0.048, Cohen’s d = 0.25) (Fig. 5A) and incongruent trials (*p* = 0.029, Cohen’s d = 0.28) (Fig. 5B).

Similarly, MANT RT analysis including load as a factor revealed a significant main effect of time [F(1,1208) = 13.540, *p* < 0.001] and load [F(2,1208) = 28.867, *p* < 0.001]. Quicker reaction times were observed following omega blend treatment on high load trials only (*p* = 0.035, Cohen’s d = 0.27) (See Fig. 5C).

#### TST

There were no significant time or treatment-related effects for TST accuracy or RT.

#### 0-back task

There were no significant time or treatment-related effects for 0-back accuracy or RT.

### Mood/emotion regulation

ANOVA output for all mood and emotion regulation outcomes can be found in Table 6. For emotion regulation, while there were no significant treatment-related effects, there was a trend for a main effect of time [F(1,124) = 2.965, *p* = 0.088] for reappraisal, however subsequent pairwise comparisons revealed no significant within-treatment changes over time. No significant effects were observed for PANAS positive affect or negative affect.

**Table 6:** – Mood/emotion regulation data – summary statistics from ANOVA analyses

Measure	N	Omega blend				Control						
		Baseline		16 weeks		Baseline		16 weeks		ANOVA Effects [(df) = F, p value]		
		Mean	SE	Mean	SE	Mean	SE	Mean	SE	Treatment	Time	Treatment x Time
*PANAS (out of 50):*												
Positive affect	64	40.2	1.00	41.5	1.00	41.2	1.00	42.2	1.00	(1,124) = 0.690, .408	(1,124) = 1.382, .242	(1,124) = 0.012, .913
Negative affect	64	25.8	1.36	28.1	1.36	27.3	1.36	29.1	1.36	(1,124) = 0.888, .348	(1,124) = 2.232, .138	(1,124) = 0.043, .836
*ERQ:*												
Suppression	64	11.1	0.43	10.7	0.43	11.3	0.43	10.5	0.43	(1,124) = 0.000, 1.000	(1,124) = 1.943, .166	(1,124) = 0.264, .609
Reappraisal	64	19.8	0.50	20.8	0.50	19.9	0.50	20.6	0.50	(1,124) = 0.009, .925	**(1,124) = 2.965, .088†**	(1,124) = 0.119, .731

Reported values are estimated marginal means; †non-significant trend 0.1 > *p* > 0.05, *significant effect *p* < 0.05

### EEG

ANOVA output for all EEG outcomes can be found in Table 7.

**Table 7:** - EEG data – summary statistics from ANOVA analyses

Measure	N	Omega blend				Control						
		Baseline		16 weeks		Baseline		16 weeks		ANOVA Effects [(df) = F, p value]		
		Mean	SE	Mean	SE	Mean	SE	Mean	SE	Treatment	Time	Treatment x Time
*0-back: PSD*												
Frontal:												
Delta	36	13.72	2.94	9.72	3.02	15.74	2.94	8.16	3.02	(1,64) = 0.048, .827	(1,64) = 1.224, .273	(1,64) = 0.003, .954
Theta	37	1.66	0.18	1.09	0.19	1.94	0.17	1.53	0.17	(1,66) = 4.165, .045*	(1,66) = 7.277, .009*	(1,66) = 0.202, .654
Alpha	37	0.77	0.08	0.53	0.08	0.81	0.08	0.66	0.08	(1,66) = 1.124, .293	(1,66) = 6.023, .017*	(1,66) = 0.261, .611
Beta	36	0.25	0.04	0.21	0.04	0.29	0.04	0.28	0.04	(1,64) = 2.134, .149	(1,64) = 0.326, .570	(1,64) = 0.196, .659
Parietal:												
Delta	37	22.2	1.97	21.7	2.03	19.3	1.97	16.7	2.03	**(1,66) = 3.797, .056†**	(1,66) = 0.635, .428	(1,66) = 0.284, .596
Theta	38	3.87	0.46	3.94	0.48	4.17	0.46	3.65	0.45	(1,68) = 0.000, .987	(1,68) = 0.254, .616	(1,68) = 0.396, .531
Alpha	36	2.73	0.46	3.01	0.47	2.85	0.44	2.96	0.46	(1,64) = 0.006, .939	(1,64) = 0.186, .668	(1,64) = 0.033, .856
Beta	37	0.53	0.06	0.53	0.06	0.47	0.06	0.47	0.06	(1,66) = 0.921, .341	(1,66) = 0.001, .982	(1,66) = 0.000, .986
*0-back: ERP*												
Parietal:												
P300 Amplitude:												
Target	38	15.80	1,21	13.60	1.29	14.50	1.115	13.70	1.15	(1,140) = 0.178, .673	(1,140) = 2.543, .113	(1,140) = 0.000, .990
Non-target	38	10.10	1.21	9.50	1.29	12.10	1.15	10.20	1.15			
P300 Latency:												
Target	38	351	10.80	338	11.50	341	10.30	356	10.30	(1,140) = 0.155, .695	(1,140) = 0.005, .947	(1,140) = 2.358, .127
Non-target	38	323	10.80	312	11.50	317	10.30	325	10.30			
N200 Amplitude:												
Target	38	-4.07	0.85	-5.38	0.90	-3.69	0.81	-1.66	0.81	(1,140) = 9.807, .002*	(1,140) = 0.193, .661	(1,140) = 4.295, .040*
Non-Target	38	-2.68	0.85	-3.62	0.90	-1.71	0.81	-1.07	0.81			
N200 Latency:												
Target	38	202	4.23	196	4.48	193	4.01	198	4.01	(1,140) = 0.697, .405	(1,140) = 0.115, .735	(1,140) = 0.643, .424
Non-Target	38	187	4.23	190	4.48	187	4.01	189	4.01			
*Resting (eyes open): PSD*												
Frontal:												
Delta	38	9.46	1.07	5.72	1.10	9.11	1.04	7.17	1.07	(1,68) = 0.237, .628	(1,68) = 6.900, .011*	(1,68) = 0.713, .402
Theta	38	1.44	0.15	1.00	0.16	1.65	0.15	1.33	0.15	**(1,68) = 3.201, .078†**	(1,68) = 5.954, .017*	(1,68) = 0.167, .684
Alpha	38	0.72	0.10	0.50	0.10	0.87	0.09	0.72	0.10	**(1,68) = 3.863, .054†**	**(1,68) = 3.552, .064†**	(1,68) = 0.152, .698
Beta	37	0.24	0.05	0.21	0.05	0.30	0.05	0.27	0.05	(1,66) = 1.503, .225	(1,66) = 0.269, .606	(1,66) = 0.003, .959
Parietal:												
Delta	38	18.4	1.49	16.7	1.53	16.3	1.45	14.4	1.49	(1,68) = 2.158, .146	(1,68) = 1.540, .219	(1,68) = 0.004, .948
Theta	38	3.26	0.39	3.32	0.40	3.44	0.39	3.18	0.38	(1,68) = 0.002, .961	(1,68) = 0.074, .786	(1,68) = 0.175, .677
Alpha	36	2.29	0.54	2.97	0.56	3.22	0.53	3.02	0.54	(1,64) = 0.866, .356	(1,64) = 0.186, .668	(1,64) = 0.667, .417
Beta	37	0.43	0.05	0.48	0.06	0.43	0.05	0.42	0.06	(1,66) = 1.015, .317	(1,66) = 0.000, .985	(1,66) = 0.008, .931
*Resting (eyes closed): PSD*												
Frontal:												
Delta	37	12.29	1.29	9.43	1.32	10.86	1.29	8.01	1.32	(1,66) = 1.203, .277	(1,66) = 4.785, .032*	(1,66) = 0.000, .998
Theta	35	1.52	0.14	0.92	0.14	1.56	0.13	1.27	0.13	(1,62) = 1.953, .167	(1,62) = 9.885, .003*	(1,62) = 1.238, .270
Alpha	37	1.07	0.15	0.64	0.15	1.02	0.15	1.15	0.15	(1,66) = 2.157, .147	(1,66) = 0.958, .331	**(1,66) = 3.362, .071†**
Beta	35	0.20	0.02	0.13	0.02	0.21	0.02	0.19	0.02	**(1,62) = 3.414, .069†**	(1,62) = 4.568, .037*	(1,62) = 1.034, .312
Parietal:												
Delta	38	20.50	2.11	23.10	2.17	19.90	2.06	17.40	2.11	(1,68) = 2.115, .150	(1,68) = 0.000, .992	(1,68) = 1.463, .231
Theta	38	3.84	0.52	3.91	0.53	4.06	0.52	3.74	0.50	(1,68) = 0.002, .963	(1,68) = 0.068, .796	(1,68) = 0.138, .711
Alpha	38	9.14	2.03	9.70	2.09	8.21	1.98	6.81	2.03	(1,68) = 0.861, .357	(1,68) = 0.049, .825	(1,68) = 0.230, .633
Beta	37	0.57	0.06	0.58	0.06	0.49	0.06	0.47	0.06	(1,66) = 2.764, .101	(1,66) = 0.005, .946	(1,66) = 0.071, .790

Reported values are estimated marginal means; †non-significant trend 0.1 > *p* > 0.05, *significant effect *p* < 0.05

#### ERP

**Fig. 6:**
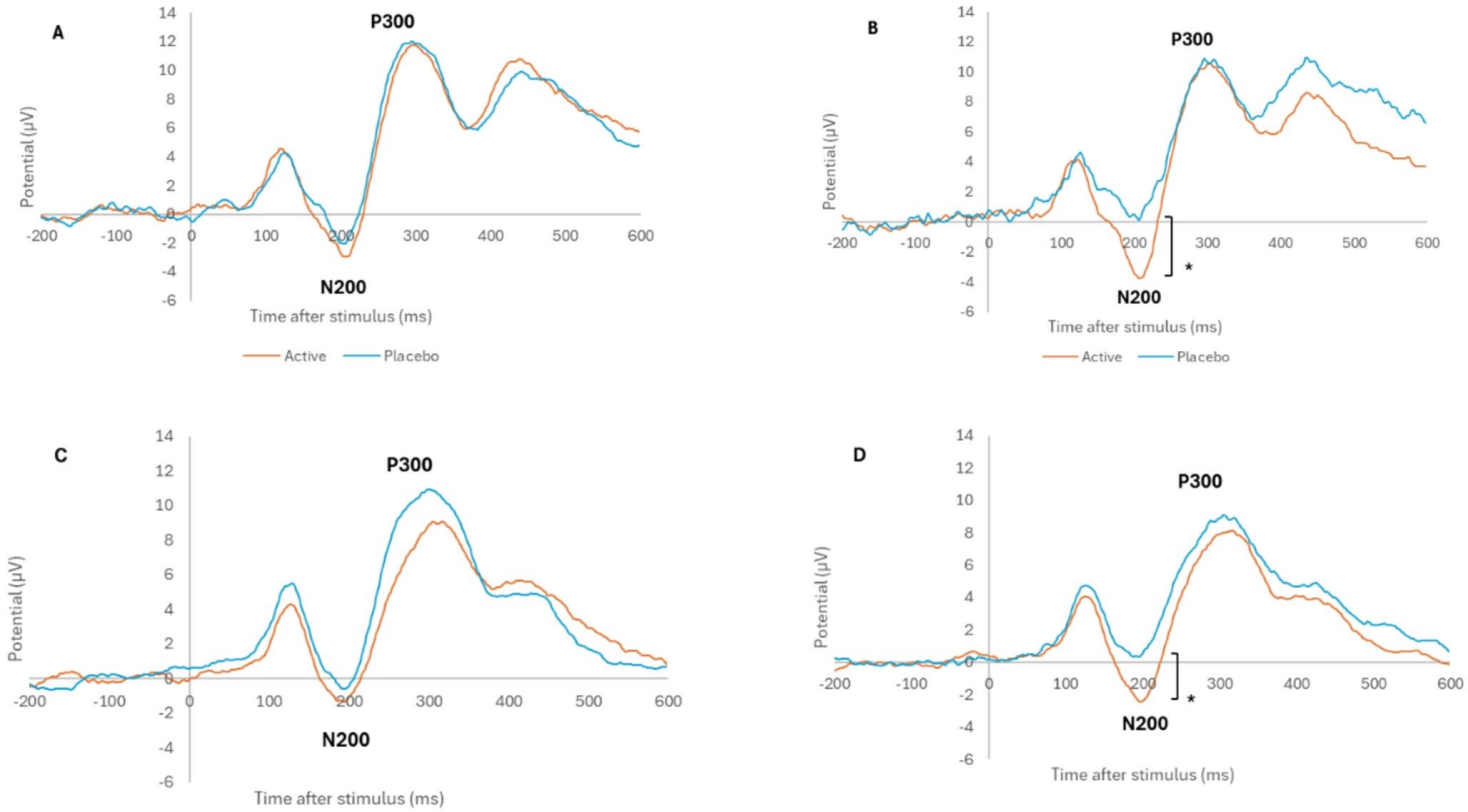
Mean parietal ERP peak amplitude (μV) for target trials at A: Baseline and B: 16 weeks and for non-target trials at C: Baseline and D: 16 weeks following omega blend or placebo treatment. *p < 0.05

EEG data was successfully collected for 38 participants (N = 19 per treatment). During the 0-back task there were no significant time or treatment-related effects for parietal P300 amplitude or latency. However, for N200 amplitude there was a significant main effect of treatment [F(1,140) = 9.81, *p* = 0.002], and time x treatment interaction [F(1,140) = 4.30, *p* = 0.040]. Target type (included as an additional factor) was also significant [F = 1,140) = 5.69, *p* = 0.018]. Pairwise comparisons revealed significantly greater N200 deflection for the omega blend group compared to the placebo group at 16 weeks for both target trials (*p* = 0.003, Cohen’s d = 0.27) (see Figs. 6A & B) and non-target trials (*p* = 0.037, Cohen’s d = 0.71) (see Figs. 6C & D). These ERP findings may suggest attentional differences in stimulus evaluation [33] following omega blend or placebo interventions.

#### PSD

**Fig. 7:**
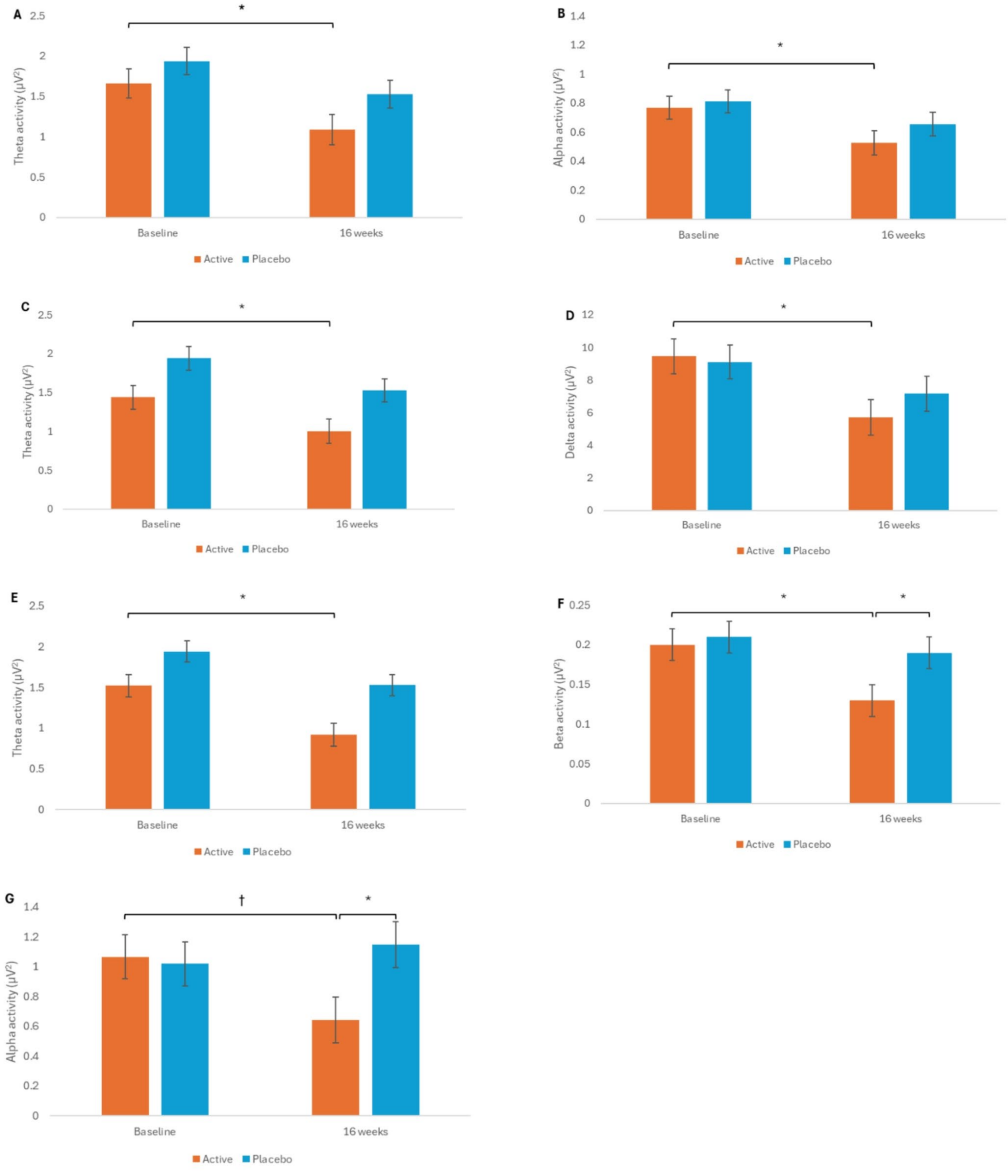
Mean frontal theta PSD activity (Panel A) and alpha PSD activity (Panel B) during 0-back task; Mean frontal theta PSD activity (Panel C) and delta PSD activity (Panel D) during rest with eyes open; Mean frontal theta PSD activity (Panel E), beta PSD activity (Panel F), and alpha PSD activity (Panel G) during rest with eyes closed. *p < 0.05, †*p* < 0.10

There were no significant PSD effects observed in the parietal brain region, with the exception of a treatment-related trend in delta activity during the 0-back task [F(1,66) = 3.80, *p* = 0.056], but this was not associated with any significant pairwise comparisons. However, in the frontal region, theta changes were observed during all tasks (the 0-back task and during rest with eyes open and closed), accompanied by some changes to delta, alpha, and beta activity. Specifically, for theta activity recorded during the 0-back task there were main effects of time [F(1,66) = 7.277, *p* = 0.009] and treatment [F(1,66) = 4.165, *p* = 0.045]. Pairwise comparisons revealed a within-treatment fall in theta in the omega blend group (*p* = 0.030; Cohen’s d = 0.77) (see Fig. 7A). Similarly, for alpha activity during 0-back, there was a main effect of time [F(1,66) = 6.02, *p* = 0.017] with a significant reduction in alpha activity observed between baseline and 16 weeks for the omega blend group (*p* = 0.040; Cohen’s d = 0.71) (see 7B).

During rest with eyes open there was a theta-related main effect of time [F(1,68) = 5.954, *p* = 0.017] and trending effect of treatment [F(1,68) = 3.201, *p* = 0.078]. Pairwise comparisons revealed a significant reduction in theta activity in omega blend group (*p* = 0.047; Cohen’s d = 0.69) (see Fig. 7C). Delta changes, as identified by a significant main effect of time [F(1,68) = 6.90, *p* = 0.011], were associated with a significant reduction in the omega blend group (*p* = 0.017; Cohen’s d = 0.43) (see Fig. 7D). Alpha changes, as identified by trending main effects of time [F(1,68) = 3.552, *p* = 0.064] and treatment [F(1,68) = 3.863, *p* = 0.055], were not associated with any significant pairwise comparisons.

During rest with eyes closed, a main effect of time for theta [F(1,62) = 9.885, *p* = 0.003] again revealed a reduction in theta in the omega blend group only (*p* = 0.004; Cohen’s d = 1.08) (see Fig. 7E). Interestingly beta activity also revealed a significant main effect of time [F(1,62) = 4.57, *p* = 0.037] and a trending main effect of treatment [F(1,62) = 3.41, *p* = 0.069], with pairwise comparisons revealing a significant reduction in the omega blend group over time (*p* = 0.031; Cohen’s d = 0.79) resulting in lower beta activity at 16 weeks compared to placebo (*p* = 0.048; Cohen’s d = 0.71) (see Fig. 7F). Similarly, a trending alpha-related time by treatment interaction [F(1,66) = 3.36, *p* = 0.071] revealed alpha activity to be lower for the omega blend group compared to placebo at 16 weeks (*p* = 0.022; Cohen’s d = 0.80) (see Fig. 7G). However, a main effect of time for delta activity [F(1,66) = 4.79,* p* = 0.032] was not accompanied by any significant pairwise comparisons.

### Regression analyses

Regression coefficients can be found in Table 8. Regression analyses were performed to test the relationships between O3I, theta PSD, and any significant cognitive outcomes. It was interesting to note that O3I trended towards being a significant positive predictor of executive function while theta activity was found to be a significant negative predictor of episodic memory. Specifically, O3I predicted ANT RT for congruent and incongruent trials (R^2^ = 0.03, *p* = 0.092, trend only) and high load trials (R^2^ = 0.08, *p* = 0.058, trend only). Theta significantly predicted RAVLT total acquisition (R^2^ = 0.33, *p* < 0.001) and RAVLT delayed recall (R2 = 0.31, *p* = 0.001). The findings suggest that higher O3I and lower theta activity may be indicative of better cognitive performance. No significant relationship was observed directly between O3I and theta activity (R^2^ = 0.02, *p* = 0.188), although the observed relationship direction was negative as might be expected (higher O3I associated with lower theta). Failure to achieve significance may simply be due to a lack of statistical power.

**Table 8:** – Regression coefficients

	Coefficient				Model	
	Beta	SE	t	Significance (p-value)	R^2^	Corrected significance^#^ (p-value)
*O3I as predictor of cognition:*						
RAVLT Total acquisition	1.85	1.08	7.61	**.090†**	0.03	.361
RAVLT Delayed recall	0.68	0.39	1.75	**.085†**	0.03	.341
ANT RT (congruent & incongruent trials)	− 17.53	7.61	− 2.30	.023*	0.03	**.092†**
ANT RT (high load trials)	− 25.76	10.22	− 2.52	.014*	0.08	**.058†**
*O3I as predictor of PSD:*						
Theta	− 0.18	0.13	− 1.35	.188	0.02	.188
*Theta as predictor of cognition:*						
RAVLT Total acquisition	− 6.24	1.49	− 4.19	< .001*	0.33	< .001*
RAVLT Delayed recall	− 2.45	0.61	− 4.01	< .001*	0.31	.001*
ANT RT (congruent & incongruent trials)	12.57	12.85	0.978	.332	< 0.01	> 0.999
ANT RT (high load trials)	34.78	17.41	2.00	**.055†**	0.09	.218

Reported values are model estimates; †non-significant trend 0.1 > *p* > 0.05, *significant effect *p* < 0.05, ^#^Bonferroni correction applied

## Discussion and overall conclusions

This double-blind randomised controlled intervention study with healthy adolescents found evidence that suggests memory and executive function benefits accompanied by changes to electrical brain activity following daily supplementation with Juice Plus+Essentials OMEGA+Blend for 16 weeks. Specifically, improvements were observed to immediate word recall (total word acquisition) and delayed word recall aspects of episodic memory. Significant improvements to reaction times (faster performance) were also observed across congruent, incongruent, and high load trials on an executive function task (MANT). Reduced theta, delta, alpha, and beta PSD activity were observed across the frontal brain region while at rest or during 0-back task performance, and ERP analysis also revealed greater N200 deflections recorded in the parietal brain region during the 0-back task.

Memory and executive function benefits outlined above were identified based on post hoc analysis of main effects of time rather than treatment related effects or interactions. As such, it should be noted that other factors such as cognitive maturation during this period may have played a role in observed improvements, and the findings warrant replication to establish an effect of treatment. It should also be highlighted that, despite employing Bonferroni as the most conservative correction to control for family-wise error, the large number of comparisons performed across outcome measures in the present study inherently raises the risk of type 1 error. Having said this, the findings in the current study are in line with previous research which has shown a positive association between omega fatty acid consumption and cognitive development in infants and children, cognitive performance in young adults, and slower age-related cognitive impairment in older adults [42]. Specifically in under 25 s, similar word recall benefits [43] and similar reaction time benefits [4, 44, 45] have been observed following omega-3 interventions. Beneficial changes to electrical brain activity following omega 3 supplementation have also been observed in children and young adults [46]. Conversely, omega 3 deficiency in adolescents with ADHD has been associated with memory deficits and high frontal theta activity [47]. Brain maturation is a process that is known to be active during adolescence and typically results in reductions in lower frequency brain activity as a result of synaptic pruning [35] that may be associated with increased cognitive efficiency [48]. As such, the observed reductions in frontal theta, delta, alpha, and beta activity in the present study may represent facilitation of brain maturation following omega FA supplementation. The observed ERP changes may also be suggestive of attentional differences in stimulus evaluation [37], although changes in N200 amplitude when responding to both targets and non-targets during the 0-back task were not corroborated by changes in the behavioural data in this case (likely due to the simplicity of this task). It should be noted, however, that EEG data is open to interpretation with discrepancy amongst experts as to what can be concluded, particularly regarding the underlying source of changes in the EEG signal measured and the localisation of effects. During cognitive task performance, multiple cognitive processes are recruited from different brain regions making interpretation of changes in neural activity difficult. Nevertheless, changes in brain activity were apparent following supplementation with the omega blend, and theta activity was observed to negatively predict memory performance following subsequent regression analyses with lower theta activity suggestive of higher memory scores, which aligns with previous research [47]. Given that EEG was only performed in a subset of the current sample and is likely underpowered, the present findings should be viewed as exploratory and hypothesis-generating, and replication in a larger adolescent cohort is recommended.

Evidence of the effects of the current omega blend intervention on mood and emotion regulation was unexpectedly lacking. We had theorised that omega blend might reasonably play a role in development of emotion regulation at this critical transition from childhood to adulthood. A non-significant statistical trend was observed for the reappraisal aspect of emotion regulation, so it may be that the current study was underpowered to fully detect such subjective emotion-related effects. The subjective measure of mood used during the study (PANAS-C) may have similarly lacked statistical power, or the use of a childhood measure of mood may have lacked sensitivity for this transitional population. Alternative measures of mood should be considered for future research such as those focussing on anxiety or depression symptoms of mental health that are known to impact this age group. In addition, the choice of placebo intervention may have further impacted statistical power. MCT oil was originally selected as a placebo comparison due to a lack of mechanistic overlap with omega FAs [49, 50] – unlike olive oil, for example, which has a similar long-chain fatty acid profile with the added complication of cognitively active phenolic compounds [51]. However, MCT oil has itself been shown to benefit mood [52] and cognitive function [53]. As such, the placebo comparison in the current trial may not have been cognitively inert. Future research may wish to consider a less bioactive placebo, although identifying such a placebo that can be matched to an omega FA blend and supplemented chronically without adverse health effects remains a challenge.

O3I was significantly increased in the omega blend group suggesting not only that study compliance was good, but also that physiological benefits of supplementation manifest in a healthy population. It should be highlighted that, although the intention was to exclude individuals approaching the recommended fruit and vegetable intake in order to capture those with sub-optimal dietary habits and greater scope for improvement, the final sample reported an average intake of roughly 4 portions per day. This may be a true reflection of the final sample, although, given that parents initially ticked to state that their child habitually consumed no more than 3 portions of fruit and veg a day, there is likely an element of overestimation by parents, which is common when completing food frequency questionnaires. Nevertheless, it is often argued that supplementation is not necessary alongside a healthy diet, however here we demonstrate that otherwise healthy adolescents still benefitted physically from the addition of a supplement to their diet. Indeed, O3I levels increased from around 75th to 95th percentile based on age-related norms [41]. O3I was also observed to tentatively predict reaction times on the ANT task (a non-significant trend), with higher O3I suggestive of faster performance. It should be noted that the supplement used here was an omega blend that also contained MUFAs omega-7 and omega-9. While we were unable to test for these in the dried blood spots, it is likely that circulating levels of these would also have increased. MUFAs from plant sources such as seed oils are known to play a role in brain function [54], and will likely have contributed to the cognitive benefits observed here, demonstrating the importance of maintaining a profile of dietary fatty acid intake.

PUFAs/MUFAs are purported to exert their beneficial effects in numerous ways. Studies have demonstrated that omega-3 PUFAs play a role in multiple aspects of brain physiology, and this biological activity can be directly related to mechanisms which may influence cognition. For example, the flexible chemical structure of omega-3 is known to increase the fluidity of neuronal membranes [55, 56]. Synaptic function is also influenced by omega-3 interaction with membrane bound proteins such as enzymes, ion channels and transporters, and glucose transporters, thus influencing signal transduction and synaptic activity [57–60]. Omega-3 PUFAs are also precursors to anti-inflammatory eicosanoids and induce the synthesis of resolvins and neuroprotectins resulting in anti-inflammatory properties [61, 62]. Omega-3 PUFAs have well established effects on endothelial function and therefore may directly impact cerebral blood flow [63]. Omega-3 PUFAs also exert neurotrophic effects by slowing the degradation of neural tissue, exerting potent anti-apoptotic effects and helping to maintain healthy axons and synaptic structures [64, 65]. To date, MUFA mechanisms have been subject to less investigation than PUFAs, possibly due to their non-essential status (i.e. they can be synthesised within the human body), but it is recognised that the positioning of the double bond plays an important role in their efficacy, with cis-MUFAs such as omega-7 and omega-9 exerting the greatest health benefits [54] via anti-inflammatory pathways, lipid metabolism, and promotion of axonal growth [54, 66]. The current study design did not permit any determination of mechanisms of action beyond the observed EEG activity and so further studies encompassing inflammatory and neurological blood markers such as IL-6 and BDNF, alongside vascular measures such as FMD are recommended.

In conclusion, findings from this double-blind randomised controlled study in healthy adolescents suggest there may be benefits to episodic memory and executive function following omega FA supplementation compared to a placebo. These cognitive benefits occurred concurrently with a range of changes in ERP & PSD brain activity, measured in a subset of participants, suggesting possible facilitation of brain maturation and/or more efficient allocation of neural resources following consumption of omega blend. The biochemical mechanisms responsible for these effects remain to be fully elucidated but may relate to anti-inflammatory, endothelial, or neurochemical pathways. This area of research warrants further research to replicate the current findings and establish mechanisms of action. Furthermore, it remains to be seen whether greater benefit would be evident in at risk populations such as those with poorer underlying diet, or those with learning deficits or attentional disorders.

## Supplementary Information

Below is the link to the electronic supplementary material.

Supplementary Material 1

## Data Availability

All data supporting the findings of this study are available within the paper.
